# Association of E-Selectin with Inflammation, Mineral and Bone Disorder, and Endothelial Dysfunction in Hemodialysis Patients

**DOI:** 10.3390/ijms27125156

**Published:** 2026-06-06

**Authors:** Crina Claudia Rusu, Diana Moldovan, Alina Potra, Dacian Tirinescu, Maria Ticala, Yuriy Maslyennikov, Alexandra Urs, Raluca Maria Pop, Madalina Ticolea, Cosmina Ioana Bondor, Ina Kacso

**Affiliations:** 1 Discipline of Nephrology, “Iuliu Hatieganu” University of Medicine and Pharmacy, 8 Victor Babes Street, 400012 Cluj-Napoca, Romania; diana.moldovan@umfcluj.ro (D.M.); alina.potra@umfcluj.ro (A.P.); tirinescu.dacian@umfcluj.ro (D.T.); cosa.maria@umfcluj.ro (M.T.); maslyennikov.yuriy@umfcluj.ro (Y.M.); alexandra.urs@elearn.umfcluj.ro (A.U.); maria.kacso@umfcluj.ro (I.K.); 2 Department of Nephrology, County Emergency Clinical Hospital Cluj, 3-5 Clinicilor Street, 400006 Cluj-Napoca, Romania; 3 Discipline of Pharmacology, Toxicology and Clinical Pharmacology, “Iuliu Hatieganu” University of Medicine and Pharmacy, 400337 Cluj-Napoca, Romania; raluca.pop@umfcluj.ro; 4 Discipline of Pathophysiology, “Iuliu Hatieganu” University of Medicine and Pharmacy, 400012 Cluj-Napoca, Romania; madalina.ticolea@umfcluj.ro; 5 Discipline of Medical Informatics and Biostatistics, “Iuliu Hatieganu” University of Medicine and Pharmacy, 6 Pasteur Street, 400349 Cluj-Napoca, Romania

**Keywords:** hemodialysis, cardiovascular complication, mineral and bone disorders, E-selectin, endothelial dysfunction, inflammation, sCD163

## Abstract

Endothelial dysfunction is an early step in atherogenesis, and adhesion molecules such as E-selectin may serve as biomarkers or therapeutic targets. This study aimed to evaluate the relationship between E-selectin and inflammatory, nutritional, and mineral–bone metabolism markers in hemodialysis (HD) patients, as well as its association with endothelial function assessed by flow-mediated dilation (FMD). We conducted a cross-sectional study including 68 HD patients (mean age 59.7 ± 12.5 years). Clinical and laboratory parameters were assessed, including inflammatory, nutritional, and mineral–bone disorder markers. Associations with E-selectin were analyzed using bivariate and multivariable models. Endothelial function was evaluated using FMD. In multivariable analysis, alkaline phosphatase (ALP) (*p* = 0.002), intact parathyroid hormone (iPTH) (*p* = 0.001), white blood cell count (WBC) (*p* = 0.015), and soluble CD163 (sCD163) (*p* = 0.042) were independently associated with E-selectin. In the subgroup with high hs-CRP values, a significant increase was observed in both E-selectin levels and adiposity tissue markers (adipose tissue mass and waist circumference). In younger patients with inflammation, E-selectin was inversely correlated with FMD (ρ = −0.64, *p* = 0.008). In conclusion, E-selectin was associated with markers of inflammation, mineral–bone disorder, and endothelial dysfunction in hemodialysis (HD) patients. Novel associations of E-Selectin with sCD163 and ALP were identified.

## 1. Introduction

Atherosclerosis is a major cause of morbidity and mortality among patients undergoing chronic hemodialysis (HD), making the investigation of its pathogenic mechanisms a priority in nephrology research [1]. Endothelial dysfunction is considered an early and critical event in atherogenesis.

In chronic kidney disease (CKD) and end-stage renal disease (ESRD), endothelial dysfunction results from the combined effects of chronic inflammation, oxidative stress, the accumulation of uremic toxins—including indoxyl sulfate, p-cresyl sulfate, advanced glycation end products, and asymmetric dimethylarginine—and impaired vascular homeostasis [2,3]. Oxidative stress further aggravates endothelial injury by reducing nitric oxide (NO) bioavailability [4] and activating proinflammatory pathways, including NF-κB signaling. In patients undergoing hemodialysis, endothelial dysfunction may be further exacerbated by dialysis-related inflammation [5]. Together, these mechanisms contribute to the increased expression of endothelial adhesion molecules, such as intercellular adhesion molecule-1 (ICAM-1), vascular cell adhesion molecule-1 (VCAM-1), E-selectin, and P-selectin [6,7,8,9], thereby promoting leukocyte adhesion and trans-endothelial migration and ultimately amplifying vascular inflammation [8,9].

Selectins are calcium-dependent lectins comprising three members: L-selectin (expressed on leukocytes), E-selectin (expressed by endothelial cells), and P-selectin (expressed by platelets) [8,10,11,12]. Soluble forms of these molecules can be measured in serum and serve as biomarkers of endothelial and platelet activation [13,14,15]. E-selectin and P-selectin are particularly upregulated during endothelial inflammation, facilitating the recruitment of leukocytes, including monocytes, neutrophils, and lymphocytes [16,17]. E-selectin also contributes to cytokine expression, such as tumor necrosis factor (TNF), and reflects the interplay between inflammation and endothelial dysfunction [18,19,20].

In the pre-dialysis stage of CKD, elevated E-selectin levels have been associated with reduced estimated glomerular filtration rate (eGFR), increased incidence of CKD, and, in end-stage renal disease (ESRD), with arteriovenous fistula stenosis [21,22,23]. In addition, in pre-dialysis CKD patients, E-selectin levels are inversely correlated with mean arterial wall thickness and with flow-mediated dilation (FMD), a non-invasive ultrasound measure of endothelial function [21,22,23,24,25]. Furthermore, E-selectin levels in ESRD may be influenced by various therapeutic interventions, including statins, vitamin D, and vitamin E, and cinacalcet [23,24,25,26,27,28,29]. Then, genetic susceptibility, particularly the Leu554Phe polymorphism of E-selectin, may contribute to variations in its circulating levels [21] and finally, ESRD-related metabolic disturbances, dietary patterns, and gut microbiota composition may modulate E-selectin levels and influence endothelial health in hemodialysis patients [30].

Therefore, this study aimed to evaluate the relationship between E-selectin and inflammatory, nutritional, and mineral–bone metabolism markers in HD patients, as well as its association with endothelial function assessed by FMD.

## 2. Results

A total of 68 patients were included in the study cohort. Of these, 58.8% were male, 14.7% had diabetes mellitus, 50% had a history of cardiovascular disease (CVD), 66.2% had hypertension, and 35.3% were active smokers. No significant differences in these baseline characteristics were observed between patients with high versus low Hs-CRP levels (< or ≥0.5 mg/dL). Baseline patient characteristics and comparison according to Hs-CRP, using the cut-off obtained from the ROC curves, are presented in Table 1. E-selectin was statistically higher in people with high Hs-CRP than in those with low Hs-CRP.

**Figure 1:**
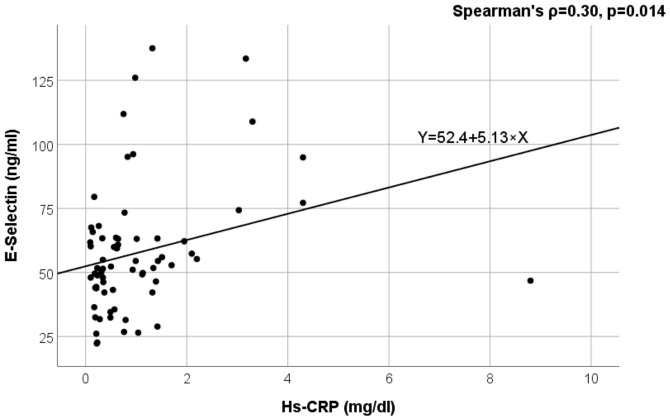
Correlation between E-selectin and Hs-CRP.

**Figure 2:**
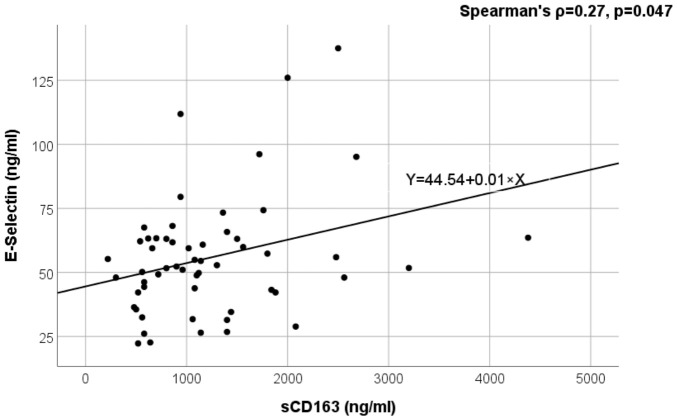
Correlation between E-selectin and sCD163.

**Figure 3:**
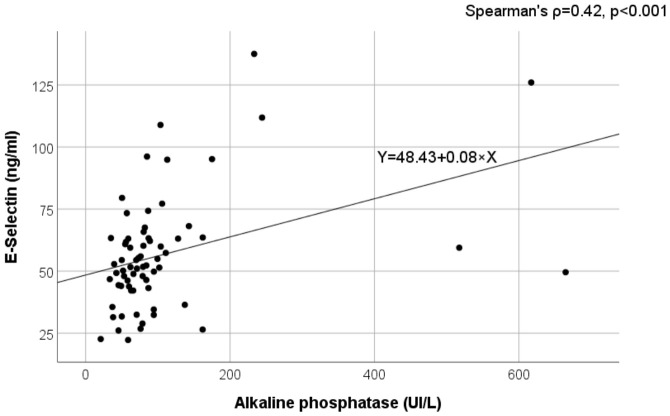
Correlation between E-selectin and alkaline phosphatase.

E-selectin levels were significantly correlated with pre-dialysis creatinine, LDL-cholesterol, cCa, ALP, Hs-CRP (Figure 1), sCD163 (Figure 2), iPTH (Figure 3), and WBC (Table 2).

The natural logarithm of E-selectin was correlated statistically significantly with the natural form of sCD163/sTWEAK, ρ = 0.28, *p* = 0.034.

In multivariable linear regression analysis with E-selectin as the dependent variable and confounding factor (age), the variables that demonstrated statistical significance were considered independent variables. Alkaline phosphatase (*p* = 0.002), iPTH (*p* = 0.001), WBC total (*p* = 0.015), and sCD163 (*p* = 0.042) remain statistically significant in the model (Table 2). Multicollinearity was assessed using variance inflation factors (VIF), and no important collinearity was observed (maximum VIF = 1.53). In a stepwise model, the variables were entered in the following order: IPTH (*p* < 0.001), ALP (*p* = 0.002), WBC (*p* = 0.005), and sCD163 (*p* = 0.012).

### Subgroup Analysis

To analyze the relationship between age and E-selectin levels under inflammatory conditions, we created two patient subgroups: one with patients < 60 years and the other with patients ≥ 60 years, both having a hs-CRP level ≥ 0.5 mg/dL. A comparison between the group aged ≥60 years and the group aged <60 years was conducted. E-selectins (ng/mL) were significantly lower [52.28 (43.18; 59.9)] in older people (≥60 years) than in younger people [63.09 (54.84; 95.63)], *p* = 0.011.

In continuation, we examined the correlations within each subgroup. In the group of patients < 60 years, E-selectin showed a significant correlation with flow-mediated dilation (FMD), LDL cholesterol, ALP, and IPTH, as shown in Table 3. However, in the age-adjusted multivariable model (enter method), only ALP remained independently associated with E-selectin levels. No significant multicollinearity was observed (maximum VIF = 1.7). In the stepwise regression analysis, iPTH (*p* = 0.005) entered the model at the first step, followed by ALP (*p* = 0.043) in the second step. These findings suggest that iPTH retains an important contribution to E-selectin variability in this subgroup; however, its independent effect appears attenuated after simultaneous adjustment for age and related covariates, likely reflecting shared variance and the reduced statistical power associated with subgroup analysis. This interpretation was supported by the analysis in the overall cohort, where iPTH remained significantly associated with E-selectin even after age adjustment. In contrast, for the group of individuals ≥ 60 years, E-selectin did not show any significant correlations with any of the measured parameters.

## 3. Discussion

In our study, E-selectin levels were associated with inflammatory markers (WBC, sCD163, and hs-CRP) as well as with markers of mineral and bone metabolism (iPTH, ALP, and corrected calcium). E-selectin was also associated with flow-mediated dilation (FMD) in younger patients with inflammation. Furthermore, in the subgroup with elevated hs-CRP levels, higher E-selectin concentrations and increased adiposity-related markers were observed. Taken together, these results suggest that E-selectin may represent a marker at the intersection of inflammation, mineral and bone metabolism, and endothelial dysfunction in HD patients.

To further explore the relationship between E-selectin and inflammatory status, we evaluated several inflammatory parameters implicated in endothelial dysfunction. Leukocytes, particularly neutrophils and monocytes, express ligands that bind E-selectin, initiating the leukocyte adhesion cascade. This interaction promotes leukocyte rolling, integrin activation, firm adhesion to the endothelium, and subsequent transmigration into tissues [20,31,32]. Uremic toxins such as indoxyl sulfate further enhance leukocyte–endothelial interactions by upregulating E-selectin expression via JNK- and NF-κB-dependent pathways [33]. In addition, the TWEAK/Fn14 axis plays a role in endothelial activation. Binding of soluble TWEAK (sTWEAK) to its receptor Fn14 induces the expression of adhesion molecules, including E-selectin and ICAM-1, thereby promoting endothelial dysfunction [32,33,34]. sTWEAK is a circulating cytokine of the TNF superfamily with angiogenic properties. Reduced circulating sTWEAK levels may result from increased binding to Fn14 or enhanced clearance mediated by sCD163, particularly under inflammatory conditions [35]. CD163 is a scavenger receptor expressed on monocytes and macrophages, which exists in a soluble form (sCD163) measurable in serum [35,36]. These interplays have been linked to endothelial dysfunction, atherosclerosis, and cardiovascular events in CKD populations [37].

In our cohort, E-selectin levels were associated with hs-CRP, leukocyte count, sCD163, and the sCD163/sTWEAK ratio, but not with sTWEAK alone. The association with leukocytes is consistent with previous studies in both CKD and non-CKD populations [17,20,21]. Notably, the relationship between E-selectin and sCD163 has been previously reported only in non-CKD populations [38,39,40]. To our knowledge, this is the first study to show an independent association between E-selectin and sCD163 in patients undergoing hemodialysis. In end-stage renal disease (ESRD), the accumulation of uremic toxins, oxidative stress, and advanced glycation end products may promote macrophage–endothelial interactions, contributing to a pro-inflammatory vascular environment [5,41]. Activated macrophages release inflammatory mediators, including tumor necrosis factor-α (TNF-α) and interleukin-1β, while activation of the metalloproteinase ADAM17/TACE mediates the shedding of membrane-bound CD163, resulting in increased circulating sCD163 levels [42]. These inflammatory stimuli activate NF-κB signaling in endothelial cells, leading to increased expression of adhesion molecules, including E-selectin, which promotes leukocyte recruitment and vascular inflammation [43]. Therefore, the observed association between sCD163 and E-selectin may reflect a macrophage–endothelial cross-talk that contributes to endothelial damage. Furthermore, the relationship between E-selectin and the sCD163/sTWEAK ratio has not been previously described. This ratio has been associated with adverse cardiovascular outcomes, including increased mortality, in both CKD and non-CKD populations [44,45,46]. It is considered a more informative biomarker than either component alone, as it reflects the balance between pro-inflammatory and regulatory pathways [47].

Regarding the relationship between E-selectin and nutritional markers, we observed that the patients with elevated inflammatory markers exhibited higher E-selectin levels and greater adiposity, as reflected by increased adipose tissue mass and waist circumference. Previous evidence indicates that visceral adipose tissue promotes endothelial activation by releasing pro-inflammatory cytokines, such as interleukin-6 (IL-6) and tumor necrosis factor-α (TNF-α), which upregulate adhesion molecules, including E-selectin [48]. In addition, E-selectin levels were inversely correlated with LDL-cholesterol. Importantly, the inverse relationship between E-selectin and LDL-cholesterol observed in our cohort reflects the well-described phenomenon of reverse epidemiology in advanced CKD and dialysis populations. Unlike in the general population, where elevated LDL-cholesterol is a major cardiovascular risk factor, multiple observational studies in CKD have demonstrated that lower LDL levels are paradoxically associated with increased mortality and cardiovascular risk [49,50]. This apparent contradiction is mainly explained by the effects of protein-energy wasting, chronic inflammation, and the comorbidity burden, which all contribute to lower circulating cholesterol levels while simultaneously increasing cardiovascular risk. In this context, lower LDL-cholesterol may represent a surrogate marker of the malnutrition–inflammation complex rather than a protective factor. Therefore, the inverse association between LDL-cholesterol and E-selectin observed in our study may indicate that patients with lower LDL levels exhibit a more pronounced inflammatory state and greater endothelial activation.

The association between endothelial dysfunction and disorders of mineral and bone metabolism is well established in chronic kidney disease [10,51,52,53]. These processes are closely interconnected, forming a bidirectional relationship. On the one hand, vascular walls exposed to hyperphosphatemia, hypercalcemia, and an elevated calcium–phosphate (Ca × PO_4_) product can act as a nidus for inflammatory stimuli, promoting the expression of adhesion molecules and contributing to endothelial dysfunction [52,54]. On the other hand, endothelial cells actively participate in vascular calcification through mechanisms such as endothelial-to-mesenchymal transition, cytokine release, and extracellular vesicle production [55]. Inhibition of endothelial–mesenchymal transition has been shown to attenuate vascular calcification [56], while damaged endothelial cells may further promote calcification through interleukin-8 secretion [57] and exosomal microRNA signaling [58]. Within this framework, it is plausible that disturbances in mineral and bone metabolism influence E-selectin levels as a marker of endothelial activation. Previous studies have demonstrated associations between soluble E-selectin and markers such as serum phosphate, the Ca × PO_4_ product, and intact parathyroid hormone [10,22,52,53]. Moreover, E-selectin has been identified as a risk factor for the progression of vascular calcification in patients undergoing chronic hemodialysis [48,49,50,51]. In our study, we observed a strong and consistent association between E-selectin levels and markers of mineral and bone metabolism. Higher levels of corrected calcium, iPTH, and alkaline phosphatase were associated with increased E-selectin concentrations, both in the overall cohort and across subgroup analyses. Notably, iPTH and alkaline phosphatase emerged as the most important determinants of E-selectin levels in our research.

ALP is a well-established independent predictor of hospitalization, cardiovascular events, and mortality in HD patients, often outperforming iPTH alone [59,60,61]. Elevated ALP levels reflect increased bone turnover but are also associated with systemic inflammation [62]. From a mechanistic perspective, tissue-nonspecific alkaline phosphatase (TNAP) contributes to vascular calcification through regulation of the balance between inorganic phosphate (Pi) and inorganic pyrophosphate (PPi). Under physiological conditions, PPi inhibits ectopic mineralization by preventing hydroxyapatite crystal formation within the vascular wall [63]. TNAP promotes calcification by hydrolyzing PPi into Pi, thereby reducing anti-calcification defense and increasing phosphate availability for mineralization [64,65]. The resulting increase in the Pi/PPi ratio favors calcium phosphate crystal deposition. It promotes the osteogenic trans-differentiation of vascular smooth muscle cells, supporting the concept of vascular calcification as an active, bone-like process [66]. In turn, vascular calcification may contribute to endothelial activation through increased arterial stiffness and local inflammation. Exposure to abnormal mechanical stress and calcium phosphate crystals may induce endothelial expression of adhesion molecules, including VCAM-1, ICAM-1, and E-selectin, thereby promoting leukocyte recruitment and vascular inflammation [67]. Bone-specific ALP (bALP) is a more sensitive marker of bone turnover and may provide superior prognostic information compared to total ALP or iPTH; however, its routine use is limited by availability and cost [68,69].

Taken together, these findings suggest the existence of a pathophysiological axis linking mineral and bone metabolism disorders to endothelial activation, with E-selectin serving as a potential biomarker of this interaction.

Regarding the relationship between E-selectin and age, no significant association was observed in the overall cohort. Available data in hemodialysis patients are limited, but existing evidence indicates that E-selectin is not significantly influenced by age, consistent with our overall findings [70]. But, among older people (≥60 years), we observed significantly lower E-selectin levels. These results were unexpected and may reflect age-related endothelial alterations, including a reduced ability to respond to chronic inflammatory stimuli in the context of immune-senescence and prolonged exposure to the uremic milieu [71,72]. A potential survivor effect may also contribute, as older patients on chronic hemodialysis may represent a selected subgroup with a distinct inflammatory and vascular profile [72].

In the overall cohort, we did not observe a significant association between E-selectin levels and flow-mediated dilation or nitroglycerin-mediated dilation. However, among younger patients with an inflammatory profile, E-selectin was inversely associated with FMD. Owing to the limited size of this subgroup, these results should be interpreted as hypothesis-generating rather than confirmatory. Our result is consistent with previous observations in pre-dialysis CKD populations but has not been well documented in patients undergoing dialysis [26]. FMD is a non-invasive, ultrasound-based technique used to assess endothelial function by measuring vasodilation in response to increased blood flow. Reduced FMD reflects impaired vascular reactivity and is associated with a higher risk of cardiovascular complications in dialysis patients [34,73]. Importantly, FMD and E-selectin provide complementary information regarding endothelial status. While FMD reflects the endothelium’s functional capacity to regulate vascular tone, E-selectin is a biochemical marker of endothelial activation and inflammation [26]. Collectively, these findings indicate that in patients with a specific metabolic context, E-selectin may serve as a marker of functional endothelial impairment. These results support the concept of personalized medicine.

This study has several strengths. First, it highlights the possible position of E-selectin at the intersection of multiple processes, including inflammation, bone and mineral metabolic disorders, and endothelial dysfunction in hemodialysis patients. Second, we identified key factors associated with E-selectin levels, particularly leukocyte count, iPTH, and alkaline phosphatase, which are clinically accessible markers. Third, we report novel associations, including the relationship between E-selectin and sCD163, that have not been previously described in hemodialysis populations. Fourth, our study results support the concept of reverse epidemiology by identifying an inverse association between E-selectin and LDL-cholesterol. Fifth, subgroup analyses revealed context-dependent associations such as the relationship of E-selectin with flow-mediated dilation, observed only in young patients with inflammation, supporting a personalized medicine approach.

Our study had some limitations. First, its cross-sectional and observational design precludes any inference of causal relationships between E-selectin and the investigated parameters. Second, the relatively small sample size may limit the statistical power and generalizability of the findings. Our results are “hypothesis-generating” rather than “confirmatory. Third, the absence of a control group restricts comparisons with non-dialysis populations. Finally, although multiple subgroup analyses were performed, these results should be interpreted with caution due to potential residual confounding. Larger, prospective studies are needed to validate these findings and to further clarify the role of E-selectin in the pathophysiology of endothelial dysfunction in hemodialysis patients.

## 4. Materials and Methods

### 4.1. Patients

We conducted a single-center, cross-sectional observational study of a selected cohort of patients with HD at a chronic dialysis center in Cluj-Napoca. The inclusion criteria were prevalent HD patients aged ≥18 years with a maintenance HD duration of at least 6 months (HD age) and without residual renal function. We excluded patients with acute inflammatory processes, terminal neoplasia, previous renal transplantation, immunosuppressive treatment, and active hepatitis or with liver test changes. 68 patients met the inclusion and exclusion criteria and agreed to participate in this study. Patient demographic and clinical data, duration of maintenance HD, and comorbidities (diabetes, hypertension, smoking, statin treatment), as well as age, weight, height, systolic blood pressure (SBP), and diastolic blood pressure (DBP) (pre-dialysis values) at enrollment, were obtained from medical records. Pulse pressure (PP) was calculated with the following formula: (PP) = SBP − DBP (mmHg). We use many anthropometric measurements. To calculate body mass index (BMI), we used the formula BMI = weight (kg)/height^2^ (m^2^). The other anthropometric parameters were measured by bioimpedance using the Body Composition Monitor, a certified device (manufactured by Fresenius Medical Care, Bad Homburg, Germany) that provided body composition as follows: lean tissue mass (LTM) (kg) and adipose tissue mass (ATM) (kg) [74].

### 4.2. Laboratory Parameters

All biochemical analyses were performed after an overnight fast between 7:00 and 9:00 AM, always during a midweek dialysis-free day. Initial measurements: serum electrolytes, albumin, uric acid, ferritin, lipid profile (total cholesterol, triglycerides [TG], and HDL-cholesterol), high-sensitive C-reactive protein (Hs-CRP), alkaline phosphatase (ALP), intact parathyroid hormone (iPTH), and transaminases. For WBC determination, we used spectrophotometry; for hs-CRP, immunoturbidimetry; for iPTH, electrochemiluminescence; and for ALP, spectrophotometry. Pre-dialysis and post-dialysis urea levels were used to calculate Kt/V as a marker of dialysis efficiency. Serum calcium was corrected (cCa) for albumin according to the formula: cCa (mg/dL) = serum calcium (mg/dL) + 0.8 × (4.0 − serum albumin (g/dL). In addition, we measured other inflammatory markers, including soluble tumor necrosis factor-like weak inducer of apoptosis (sTWEAK), sCD163, and E-selectin, using an enzyme-linked immunosorbent assay (ELISA) with commercially available kits (R&D Systems, Minneapolis, MN, USA). The minimum detection limit was for sTWEAK—10 pg/mL, and the intra- and inter-assay coefficients of variation were 7.9% and 9.1%, respectively; for sCD163, the minimum detectable level was 0.613 ng/mL, and the intra- and inter-assay coefficients of variation were 5.1 and 3.5%, and for E Selectin the minimum detectable level was 0.1 ng/mL and the intra- and inter-assay coefficients of variation were 5.4% and 7.9%.

As a summary of the direct study variables, we mention a comprehensive set of biomarkers that may influence E-selectin levels in hemodialysis patients. Inflammatory markers included hs-CRP, WBC, ferritin, sCD163, sTWEAK, and the sCD163/sTWEAK ratio. Anthropometric and body composition markers included: BMI, LTM, ATM, WC, and TST. Serum nutritional markers included pre-dialysis albumin as an indicator of protein status, and total cholesterol and LDL-cholesterol were also analyzed.

### 4.3. Dialysis Prescription

All patients included in the study received conventional HD three times per week, with each session lasting 4 to 5 h. Only 5 patients had 5 h sessions; the remaining patients had 4 h sessions. Dialysis was performed using disposable synthetic polysulfone dialyzers, with heparin as the standard anticoagulant. A nephrologist guided the dialysis prescription to achieve a Kt/V > 1.4. Medications for disorders related to mineral and bone metabolism, anemia, hypertension, and cardiovascular diseases were administered according to the guidelines in effect at the time of the study. However, the specific doses for these drug classes were not recorded. Antihypertensive treatment was prescribed for patients with persistent blood pressure readings above 150/90 mmHg, whether measured post-dialysis or during inter-dialysis periods. Ultrafiltration during HD was performed according to the patient’s dry weight.

Vascular measurements to determine flow-mediated vasodilation were performed by ultrasound, as described in another of our articles [75].

### 4.4. Statistical Analysis

Variables were described for both the overall cohort and the subgroups using descriptive statistics, such as means, standard deviations, medians, and quartiles for normally and non-normally distributed variables; and absolute and relative (percentages) frequencies for categorical variables. Independent subgroups were compared using Student’s *t*-test when subgroup sizes were large (over 30 subjects) or when smaller subgroups had normally distributed variables; otherwise, the Mann–Whitney U test was used. Frequencies across groups were compared using the chi-square test.

Associations between the variable of interest and other continuous quantitative variables were assessed using Spearman’s correlation coefficient and multivariable regression analyses, conducted in both the overall cohort and in subgroups.

To identify predictors of the variable of interest, multiple linear regression models were constructed, including confounding variables and predictors that were statistically significant in the correlation analysis. Multicollinearity was assessed, variables that did not meet this criterion were excluded from the predictive models, and maximum VIF of the final enter model was reported. For subgroups analysis, to reduce the risk of overfitting, the number of covariates included in the multivariable model was limited relative to the sample size. Covariates were selected based on biological plausibility and prior evidence, while stepwise regression analysis was additionally performed to identify the variables most strongly associated with E-selectin levels. A significance level of α = 0.05 was applied. Statistical analyses were conducted using SPSS version 25.

## 5. Conclusions

E-selectin may be involved in several pathophysiological processes, including inflammation, disorders of mineral and bone metabolism, and endothelial dysfunction in hemodialysis patients. In this study, higher E-selectin levels were associated with inflammatory markers (leukocyte count, hs-CRP, and sCD163), as well as markers of mineral and bone metabolism (ALP, iPTH, and corrected calcium). An inverse relationship between E-selectin and LDL cholesterol was also observed, which may be compatible with the phenomenon of reverse epidemiology described in advanced chronic kidney disease (CKD). Subgroup analyses suggested context-specific associations, including a relationship between E-selectin and endothelial function, assessed by flow-mediated dilation (FMD), in younger patients with inflammation. The observed associations with clinically accessible parameters, such as ALP and white blood cell (WBC) count, may indicate a possible role of E-selectin in risk stratification. However, given the cross-sectional design, these findings should be interpreted with caution, and further prospective studies are needed to better clarify these associations.

## Figures and Tables

**Table 1:** Patient Characteristics and Comparison by Hs-CRP Using the ROC-Derived Cut-Off [mean ± standard deviation or median (25th–75th percentile)].

Parameters	All Patients (*n* = 68)	Hs-CRP < 0.5 mg/dL (*n* = 28)	Hs-CRP ≥ 0.5 mg/dL (*n* = 40)	*p*
Male, *n* (%)	40 (58.8)	17 (60.7)	23 (57.5)	0.791
Age (years)	59.71 ± 12.48	57.36 ± 13.02	61.35 ± 11.98	0.196
HD duration (months)	70.97 ± 48.51	77.82 ± 52.85	66.18 ± 45.29	0.462
FMD (%)	7.49 ± 7.65	7.25 ± 6.78	7.66 ± 8.31	0.836
NMD (%)	9.51 ± 9.55	9.33 ± 6.67	9.63 ± 11.13	0.977
Waist circumference (cm)	96.42 ± 15.79	91.81 ± 13.52	99.53 ± 16.59	0.049
Triceps skinfold thickness (mm)	3 (2.5; 4)	3 (2; 4)	3.75 (3; 4)	0.082
SBP (mmHg)	141.5 ± 20.14	137.96 ± 19.57	143.98 ± 20.41	0.229
DBP (mmHg)	76.5 (70; 80)	76.5 (69; 80)	78 (70; 80)	0.751
kt/v	1.59 ± 0.33	1.6 ± 0.39	1.57 ± 0.28	0.400
Triglycerides (mg/dL)	136.5 (94.36; 184.95)	130.19 (82.9; 164)	139 (97.05; 237)	0.276
LDL-cholesterol (mg/dL)	101.64 ± 39.1	108.73 ± 38.87	96.68 ± 38.97	0.246
HDL-cholesterol (mg/dL)	40.66 (30.54; 47.73)	46.2 (32.93; 53.57)	34.95 (29.75; 45.65)	0.031
Corrected Calcium (mg/dL)	8.81 ± 0.65	8.61 ± 0.57	8.94 ± 0.67	0.036
Phosphorus (mg/dL)	4.89 (4.09; 6.15)	4.98 (4.04; 5.92)	4.87 (4.19; 6.4)	0.667
Alkaline phosphatase (UI/L)	78.7 (56.42; 100.73)	62.5 (51.12; 88.18)	84 (63.82; 108.44)	0.049
Intact parathormone (pg/mL)	362 (169.2; 882.8)	348.65 (169.2; 732.9)	381.95 (192.15; 1129.5)	0.681
Fasting glucose (mg/dL)	95.5 (88.1; 115.15)	91.53 (83.75; 103.5)	99.33 (91.43; 119.62)	0.064
Pre-dialytic creatinine (mg/dL)	8.72 ± 2.49	8.46 ± 2.41	8.9 ± 2.56	0.480
Serum albumin (g/L)	3.93 ± 0.25	3.95 ± 0.29	3.92 ± 0.22	0.603
Hs-CRP (mg/dL)	0.62 (0.25; 1.32)	0.23 (0.18; 0.34)	1.08 (0.76; 1.61)	
Ferritin (ng/mL)	590.56 ± 324.98	464.14 ± 268.99	681.32 ± 334.36	0.006
WBC (no/mmc)	6235 (5415; 7210)	5710 (5195; 6415)	6605 (5695; 7955)	0.001
Hemoglobin (g/dL)	11.38 ± 1.12	11.5 ± 0.81	11.29 ± 1.3	0.411
Serum Bicarbonate (mmol/L)	20.95 (18.2; 24.9)	21.05 (18.7; 24.85)	20.9 (18.2; 24.95)	0.592
sCD163 (ng/mL)	107 (630; 1530)	700 (570; 1070)	1360 (940; 1840)	0.001
sTWEAK (pg/mL)	3661.04 (3103.24; 4611.07)	3935.09 (3478.18; 4374.18)	3513.41 (2769.48; 4721.51)	0.357
sCD163/sTWEAK (ng/pg)	0.27 (0.16; 0.43)	0.19 (0.14; 0.32)	0.3 (0.19; 0.55)	0.047
E-Selectin (ng/mL)	52.55 (43.92; 63.28)	48 (35.48; 57.57)	58.38 (49.52; 73.82)	0.006
BMI (kg/m^2^)	27.71 (23.52; 30.66)	25.89 (23.28; 29.54)	28.62 (26.05; 31.72)	0.054
LTM (kg)	31.98 ± 8.12	32.44 ± 8.17	31.67 ± 8.18	0.716
ATM (kg)	41.11 ± 16.42	35.76 ± 14.61	44.63 ± 16.78	0.035

LTM—lean tissue mass; Hs-CRP—high-sensitivity C-reactive protein; HD—hemodialysis; FMD—flow-mediated dilatation; NMD—nitroglycerin-mediated dilatation; SBP—systolic blood pressure; BMI—body mass index; DBP—diastolic blood pressure; sCD163—soluble CD163; WBC—white blood cells; sTWEAK—soluble tumor necrosis factor-like weak inducer of apoptosis; ATM—adipose tissue mass; *p* was calculated between columns 2 and 3 of Table 1.

**Table 2:** Correlation between E-Selectin and inflammatory, nutritional markers, mineral and bone disorders markers, NMD and FMD, and other parameters in the total group.

	Bivariate Analysis		Multivariable Analysis *
Parameters	Spearman Correlation Coefficient (*n* = 68)	*p*	*p*
Age (years)	−0.21	0.086	
FMD (%)	−0.18	0.155	
NMD (%)	0.04	0.785	
Waist circumference (cm)	0.02	0.859	
Triceps skinfold thickness (mm)	−0.06	0.632	
LDL-cholesterol (mg/dL)	−0.25	0.040	0.526
Cholesterol total (mg/dL)	−0.19	0.123	
Corrected Calcium (mg/dL)	0.25	0.040	0.648
Phosphorus (mg/dL)	0.21	0.083	
Alkaline phosphatase (UI/L)	0.42	<0.001	0.002
Intact parathormone (pg/mL)	0.30	0.014	0.001
Pre-dialytic creatinine (mg/dL)	0.29	0.017	0.302
Serum albumin (g/L)	−0.03	0.797	
Hs-CRP (mg/dL)	0.30	0.014	0.945
WBC (no/mmc)	0.38	0.001	0.015
sCD163 (ng/mL)	0.27	0.047	0.042
sTWEAK (pg/mL)	−0.15	0.247	
sCD163/sTWEAK (ng/pg)	0.22	0.098	
BMI (kg/m^2^)	−0.01	0.925	
LTM (kg)	0.08	0.545	
ATM (kg)	0.00	0.997	

LTM—lean tissue mass; Hs-CRP—high-sensitivity C-reactive protein; FMD—flow-mediated dilatation; NMD—nitroglycerin-mediated dilatation; WBC—white blood cells; sTWEAK—soluble tumor necrosis factor-like weak inducer of apoptosis; BMI—body mass index; sCD163—soluble CD163; ATM—adipose tissue mass; * the model was adjusted for Age.

**Table 3:** Correlation of e-Selectin levels with other quantitative parameters in the subgroup of people with age ≥ 60 years and the subgroup with age < 60 years, only in people with high Hs-CRP (Hs-CRP ≥ 0.5 mg/dL).

Parameter	Age < 60 Years (*n* = 19)			Age ≥ 60 Years (*n* = 21)	
	Bivariate Analysis		Multivariable Analysis	Bivariate Analysis	
	Spearman Correlation Coefficient	*p*	*p*	Spearman Correlation Coefficient	*p*
FMD (%)	−0.64	0.008		0.04	0.867
LDL-cholesterol (mg/dL)	−0.48	0.038		−0.20	0.391
Alkaline phosphatase (UI/L)	0.84	<0.001	0.014	0.23	0.312
Intact parathormone (pg/mL)	0.62	0.004		0.08	0.737

Hs-CRP—high-sensitivity C-reactive protein; LDL-cholesterol; FMD—flow-mediated dilatation.

## Data Availability

The research data supporting this study’s findings are not publicly available. Further inquiries can be directed to the corresponding authors.

## Abbreviations

The following abbreviations are used in this manuscript:

Hs-CRP	high-sensitivity C-reactive protein
HTA	hypertension
HD	hemodialysis
ALP	alkaline phosphatase
iPTH	intact parathormone
FMD	flow-mediated dilatation
NMD	nitroglycerin-mediated dilatation
SBP	systolic blood pressure
DBP	diastolic blood pressure
PP	pulse pressure
WBC	white blood cells
sCD163	soluble CD163
sTWEAK	soluble tumor necrosis factor-like weak inducer of apoptosis
BMI	body mass index
LTM	lean tissue mass
ATM	adipose tissue mass
CKD	chronic kidney disease
CVH	cardiovascular history
